# Comparative evaluation of efficacy and safety of ophthalmic viscosurgical devices in phacoemulsification [ISRCTN34957881]

**DOI:** 10.1186/1471-2415-5-17

**Published:** 2005-07-15

**Authors:** Rasik B Vajpayee, Kamna Verma, Rajesh Sinha, Jeewan S Titiyal, RM Pandey, Namrata Sharma

**Affiliations:** 1 Rajendra Prasad Centre for Ophthalmic Sciences, All India Institute of Medical Science, New Delhi, India; 2 Department of Biostatistics, All India Institute of Medical Science, New Delhi, India

**Keywords:** ophthalmic viscosurgical device, phacoemulsification, corneal endothelium

## Abstract

**Background:**

Various ophthalmic viscosurgical devices (OVD) are used to perform phacoemulsification and other intraocular surgeries. We performed a study to compare the efficacy and safety of three ophthalmic viscosurgical devices that are routinely used in phacoemulsification.

**Methods:**

Fifty-six patients of immature senile cataract with hard nucleus (grade 3 and 4) who underwent phacoemulsification were included. Depending upon the type of OVD, patients were randomly allocated into three groups; group 1 (n = 19), Viscoat^® ^was used; group 2 (n = 19), Healon GV^® ^was used; group 3 (n = 18), Healon 5^® ^was used. Parameters evaluated were uncorrected and best corrected visual acuity, specular microscopy, intraocular pressure and pachymetry both preoperatively and postoperatively on day 1, 1 week, 1 month and 3 months and development of any complication both intraoperative and postoperative were also noted.

**Results:**

The mean increase in central corneal thickness was 15.17% (group 1); 17.26% (group 2) and 16.21% (group 3) on first postoperative day and was comparable in the three groups. The density of endothelial cells decreased postoperatively (day 1) by 12.54% (group 1), 13.76% (group 2) and 13.06% (group 3) and was comparable. The mean preoperative intraocular pressure in groups 1, 2 and 3 were 13.3 ± 2.0, 14.0 ± 2.2 and 13.2 ± 3.2 mmHg respectively, which changed to 16.0 ± 4.7, 12.2 ± 4.7 and 12.3 ± 4.8 respectively on first postoperative day and the change in intraocular pressure was significantly higher in group 1 (1 vs 2 & 1 vs 3; p = 0.02; oneway ANOVA).

**Conclusion:**

Viscoat^®^, Healon GV^® ^and Healon 5^® ^give comparable results in terms of efficacy and safety in performing phacoemulsification.

## Background

Ophthalmic Viscosurgical Devices (OVDs) have played a key role in the success of phacoemulsification. An ideal OVD is one which is able to maintain the anterior chamber during the procedure particularly capsulorhexis, phaco probe entry and initial phacoemulsification and during intraocular lens implantation, maintain mydriasis and media clarity, protect the endothelium from phaco energy and also prevent postoperative rise in intraocular pressure. Although, till date, no OVD can be considered ideal, corneal endothelial cell loss during cataract surgery has been minimized by the use of viscoelastic substances [1,2].

However, the incidence of variable degree of endothelial decompensation in patients after cataract surgery remains around 17% [3,4]. Hence, there is always the search of an ophthalmic viscosurgical device which is ideal and that can minimize damage to corneal endothelium and other intraoperative complications. Over the past few years, a partial success in this area has led to a decrease in the incidence of bullous keratopathy following cataract surgery requiring penetrating keratoplasty [5,6]. In this study, we compared the efficacy and safety of three ophthalmic viscosurgical devices namely, Viscoat^® ^(Sodium chondroitin sulfate 4.0% & sodium hyaluronate 3.0%), Healon GV^® ^(Sodium hyaluronate 1.4%) and Healon 5^® ^(Sodium hyaluronate 2.3%) in hard nucleus (grade 3 and 4).

## Methods

A randomized clinical trial was performed which comprised of 56 consecutive patients of immature senile cataract with hard nucleus, who attended ophthalmic out patient department at the Rajendra Prasad Centre for Ophthalmic Sciences, All India Institute of Medical Sciences, New Delhi, India. All patients were informed and a written consent was obtained from them to participate in the study.

Only those patients were included in the study, who were more than 40 years of age, had senile cataract, nucleus hardness of grade 3/4, not having any evidence of subluxation or pseudoexfoliation and not having any other associated ocular pathology. Patients with preoperative diagnosed glaucoma and/or IOP greater then 20 mmHg preoperatively were excluded from the study. Other exclusion criteria were intraoperative events like manual dilatation of pupil, posterior capsular rent and placement of IOL in the sulcus.

Pre-operative evaluation of the patients included the measurement of uncorrected and best corrected visual acuity (UCVA and BCVA respectively), an examination of the anterior segment of the eye under slit lamp biomicroscope, central corneal thickness and fundus evaluation through a dilated pupil. Intraocular pressure was recorded pre-operatively with a non-contact tonometer Topcon CT – 80 (Topcon America Corporation, Paramus, NJ) in all the patients. Keratometry was performed using Bausch and Lomb keratometer, while the axial length was measured using an A scan biometer (Appasamy associates, Chennai). Anterior chamber depth was recorded by Sonomed 5500 Digital A/B Scan (Latham and Phillips Ophthalmic Products Inc., Grove City, Ohio). The power of the intraocular lens was calculated in all the patients using SRK (Sanders, Retzlaff, Kraff) formula. History of any systemic illness was excluded and blood pressure was measured on admission.

Grading (0 to 4+) of the nuclear sclerosis (a combination of opalescence and yellowing) was performed on slit lamp biomicroscope. Corneal thickness was measured using Sonomed ultrasonic pachymeter (Micropach Model 200P; Latham and Phillips Ophthalmic Products Inc., Grove City, Ohio). Endothelial cell counts were performed with Topcon sp-2000P noncontact specular microscope (Topcon America Corporation, Paramus, NJ).

Randomization was done pre-operatively by the statistical random table. Surgery was performed by a single surgeon (RBV) and the operating surgeon was told on the operation table to use single viscosurgical device allocated for the patient for the entire procedure as per the randomization. Those examiners (NS, JST) who performed postoperative follow up and examination were masked and unaware about the type of viscoelastic used in a particular case.

### Surgical technique

Surgeries were performed under topical anesthesia (proparacaine 0.5%). Pupillary dilatation was achieved by a combination of topical 0.5% tropicacyl and 5% phenylephrine. A 3.2 mm clear corneal tunnel was created either superiorly or temporally depending upon the steeper axis; side port entry was made with the help of microvitreoretinal (MVR) blade. OVD was injected into the anterior chamber according to the randomization, and capsulorhexis was performed. Hydrodissection and hydrodelineation were performed to achieve free rotation of nucleus. Phacoemulsification was done using crater and chop technique by Storz protégé machine (Storz Protégé, Bausch & Lomb, NY, USA) which was followed by a thorough irrigation and aspiration of the cortical matter. Capsular bag was inflated with viscoelastic and a single piece acrylic foldable IOL (ACRYSOF^® ^SA60AT; Alcon laboratories, Fort Worth, TX) was implanted in all the patients. The OVD was completely aspirated out with the rock and roll technique. The irrigating solution and the phaco machine were similar in all the three groups.

Postoperatively, patients were prescribed 1% Prednisolone acetate and ciprofloxacin 0.3% QID each for 4 weeks and tropicamide 1% TID for 1 week.

The intraoperative parameters recorded were the type of OVD, phacoemulsification time, average ultrasonic energy used, time required to remove the viscoelastic material, maintenance of anterior chamber depth and total surgical time.

Postoperatively, all the parameters were recorded on day 1, 1 week, 1 month and 3 months using the same method and instruments.

### Statistical analysis

Data were recorded on a predesigned proforma and managed on Excel spread sheet. For quantitative variables, approximate normal distribution was assessed and subsequently mean & standard deviation (SD) was computed as summary statistics.

Preoperative values for all the parameters were statistically compared amongst the three groups. Group 1 (n = 19), comprised of eyes in which Viscoat^® ^(Sodium chondroitin sulfate 4.0% & sodium hyaluronate 3.0%) was used; Group 2 (n = 19) comprised of those in which Healon GV^® ^(Sodium hyaluronate 1.4%) was used and Group 3 (n = 18) comprised of those in which Healon 5^® ^(Sodium hyaluronate 2.3%) was used. The base line values of all the parameters were statistically similar, therefore we used one-way analysis of variance followed by Scheffe's post hoc ANOVA, if required, to compare the mean values at every line point between the three groups. Analysis of co-variance was used to compute mean and SD values at various postoperative follow up.

Repeated measures of analysis of variance were used to determine changes from the preoperative values. To compare categorical variables between the groups, Chi square or fisher's exact test, as appropriate, was used. STATA 8.0 and SPSS version 10.0 statistical softwares were used for data analysis. P value of <0.05 was considered as statistically significant.

## Results

The mean age of the patient was 68.73 ± 8.96 years and 43 (76.71%) patients were male. The three groups were comparable preoperatively in terms of age, anterior chamber depth, central corneal thickness and central endothelial cell count (Table 1). The mean preoperative uncorrected visual acuity in the three groups was 0.12 ± 0.14, 0.09 ± 0.13 and 0.11 ± 0.9 respectively, which improved to 0.51 ± 0.16, 0.61 ± 0.20 and 0.61 ± 0.20 respectively at the final follow up at 3 months. The mean preoperative best corrected visual acuity in the three groups was 0.18 ± 0.11, 0.17 ± 0.19 and 0.18 ± 0.13 respectively and this improved to 0.69 ± 0.24, 0.88 ± 0.18 and 0.79 ± 0.23 respectively at 3 months.

The total number of eyes with grade 3 nuclear sclerosis was 8 in Group 1, 11 in Group 2 and 4 in group 3. The eyes with grade 4 nuclear sclerosis were 11, 8 and 14 in group 1, group 2 and group 3 respectively.

The mean phaco energy (%) utilized was higher in Group 3 in comparison to Group 2 (p < 0.03) and Group 1 (p < 0.05) (Table 2). The total operating time was longer in Group 3 in comparison to Group 1 (p < 0.011). Time taken for removal of viscoelastic after IOL insertion in the capsular bag was higher in Group 1 when compared to Group 2 and Group 3 (Table 2). The mean increase in central corneal thickness was 15.17% in group 1; 17.26% in group 2 and 16.21% in group 3 on the first postoperative day and this change was comparable in the three groups (Table 1).

Preoperative central endothelial cell density was 2311 cells/mm^2 ^in group 1; 2359 cells/mm^2 ^in group 2 and 2526 cells/mm^2 ^in group 3 (Table 3). Postoperatively, the density of endothelial cells on day 1 decreased by 290 cells/mm^2 ^(12.54%) in group 1 [Not significant (NS)], 324 cells/mm^2 ^(13.76%) in group 2 (NS) and 330 cells/mm^2 ^(13.06%) in group 3 (NS). On comparative evaluation, there was no significant difference in the change in the endothelial count between the three groups.

The mean preoperative intraocular pressure in group 1, group 2 and group 3 were 13.3 ± 2.0, 14.0 ± 2.2 and 13.2 ± 3.2 respectively. On day 1 after surgery, the mean intraocular pressure in the three groups were 16.0 ± 4.7, 12.2 ± 4.7, and 12.3 ± 4.8 respectively. On comparative evaluation between the three groups, the rise in intraocular pressure in was found to be slightly higher in group 1 and significant statistically (1 vs 2 & 1 vs 3: p = 0.02; oneway ANOVA). At 1 week follow up, the mean intraocular pressure in the three groups were 13.8 ± 4.6, 12.0 ± 4.5 and 12.2 ± 4.7 respectively and a comparative evaluation between the groups was not significant. The mean intraocular pressure in the three groups at 3 months were 13.0 ± 3.4, 12.1 ± 3.7 and 12.3 ± 3.6 respectively (p = ns). There was no episode of peripheral extension of the capsulorhexis margin in any of the eyes. There was no evidence of any significant intraoperative or postoperative complication in any eye.

## Discussion

Optimal space maintenance during different stages of the surgical procedure is essential to minimize mechanical damage to the intraocular structures [7]. An increasing number of viscoelastics with different compositions and characteristics are now available [8]. These products differ in their space-maintaining capabilities and other properties.

In the present study, we observed that the anterior chamber was well formed in all the patients in the three groups during the entire procedure of capsulorhexis and phaco probe entry and initial phacoemulsification. This property not only helps in maintaining the chamber for better performance of the procedure but also counteracts positive vitreous pressure during the procedure.

In our study, greater number of grade 4 nucleus was seen in group 3. Hence the mean phaco energy and total surgical time was higher in this group in comparison to group 1 and group 2. However, there was no significant increase in central corneal thickness in the immediate postoperative period and also at the end of 3 months following surgery. However, pachymetry might be slightly inaccurate as it is impossible to measure exactly the same corneal area every time and measurements of several corneal parts (e.g. superior, nasal, inferior, temporal and central) should have been performed.

There was no significant difference in the change in endothelial count in eyes among the three groups. It is reported in literature that endothelial cell loss rate after phacoemulsification with IOL implantation is greater in eyes with a hard nucleus than those with a soft nucleus [9-11]. However, statistically, all the three viscosurgical devices were comparable in their endothelial protective capabilities. Ravalico G et al performed a study comparing Healon, Healon GV, Viscoat and Hymecel (2% hydroxypropylmethylcellulose) and reported that Healon GV and Viscoat are comparable in their endothelial protective property [12]. However, another study reported a superiority of Viscoat over Healon GV and Healon in terms of protection to endothelium during phacoemulsification [13]. Holzer MP et al reported in their study that Healon 5 is superior to Healon GV and Viscoat in terms of endothelial protective property [14].

Several techniques have been reported in the literature for removal of the OVDs. These include: Rock and roll technique, two-compartment technique and bimanual irrigation/aspiration technique [15]. Healon-5^® ^has been reported to be a viscoadaptive OVD as it has different functions at different flow rates. At lower flow rates it behaves as a very cohesive viscoelastic like a Healon-GV^®^. At higher flow rates, e.g. in chopping techniques, it begins to fracture and behaves similar to a dispersive viscoelastic, such as Viscoat^®^.

In the present study, greater time was required to remove the viscoelastic after IOL implantation in group 1. This suggests that Viscoat has a more retentive nature in comparison to Healon GV and Healon 5 which come out in bolus and require lesser time. Again OVD removal time was significantly higher in group 3 in comparison to group 2. This suggests that Healon 5 has a greater dispersive quality than Healon GV. Postoperatively, there was no evidence of retention of residual OVD in the anterior chamber of any eye on slit lamp biomicroscope.

There was a significant increase in the mean intraocular pressure in group 1 (although in the normal range) in comparison to group 2 (p = 0.02) and 3 (p = 0.02) on day 1 of the postoperative phase. However, at subsequent follow up visits, there was no significant difference in the mean intraocular pressure among the three groups. This transient rise in the mean intraocular pressure in group 1 suggests that although on slit lamp biomicroscopy, there was no direct evidence of retained viscosurgical device in the anterior chamber, there may be microscopic retention in the trabecular meshwork that can go undetected on routine examination. This microscopic retention can decrease the aqueous outflow transiently and result in variable increase in intraocular pressure. Subsequently, it gets drained off and the intraocular pressure returns close to the baseline value. A similar study comparing Healon 5 with Viscoat reported that the IOP in the early postoperative period was higher in the Viscoat group than in the Healon 5 group [16]. However, another study comparing Healon GV, Healon 5 and Iviz (Sodium hyaluronate 1%) concludes that although Healon 5 takes longer time for removal, there is no significant difference in the postoperative intraocular pressure [17].

## Conclusion

In the present study, we conclude that the safety and efficacy of the three viscosurgical devices namely Viscoat^®^, Healon GV^® ^and Healon 5^® ^in performing phacoemulsification is comparable. However, Viscoat^® ^can result in a mild transient rise in the intraocular pressure.

## Competing interests

The author(s) declare that they have no competing interests.

## Authors' contributions

RBV designed the study and performed surgeries. KV performed the data collection. RS wrote the manuscript. JST and NS followed up the patients. RMP performed the statistical evaluation.

All authors read and approved the final manuscript.

## Pre-publication history

The pre-publication history for this paper can be accessed here:



## Figures and Tables

**Figure 1:**
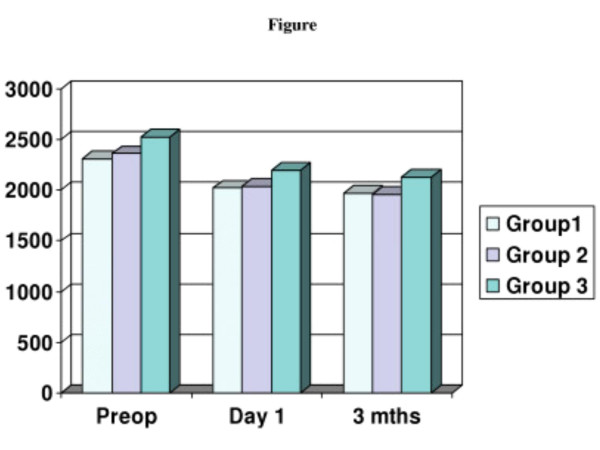
Mean Endothelial Cell Count.

**Table 1:** Pre operative and Post operative characteristics of the patients

**Parameter**	**Group 1 n = 19**	**Group 2 n = 19**	**Group 3 n = 18**	**One-way ANOVA, F-Value**	**P value**
Age	69.6 ± 9.2	65.8 ± 7.8	70.8 ± 9.9	1.58	NS
ACD	2.6 ± 0.20	2.8 ± 0.37	2.59 ± 0.45	1.97	NS
UCVA					
Pre-operative	0.12 ± 0.14	0.09 ± 0.13	0.11 ± 0.9	0.18	NS
Day one	0.28 ± 19^a^	0.39 ± 23^c^	0.37 + 0.27^b^	1.22	NS
Three month	0.51 ± 0.16^c^	0.61 ± 0.20^c^	0.61 ± 0.20^c^	1.51	NS
Repeated measure within group ANOVA	F = 15.0 P = 0.0001	F = 28.29 P = 0.0001	F = 23.5 P = 0.0001		
BCVA					
Pre-operative	0.18 ± 0.11	0.17 ± 0.19	0.18 ± 0.13	0.04	NS
Day one	0.46 ± 0.23^b^	0.69 ± 0.26^c^	0.55 ± 0.31^a^	1.70	NS
Three month	0.69 ± 0.24^c^	0.88 ± 0.18^c^	0.79 ± 0.23^c^	2.73	NS
Repeated measure within group ANOVA	F = 34.17 P = 0.0001	F = 43.8 P = 0.0001	F = 23.5 P = 0.0001		
Corneal thickness					
Pre-operative	560.7 ± 91.5	567.5 ± 44.07	576.0 ± 50.2	0.25	NS
Day one	645.8 ± 94.1^c^	665.5 ± 141.9^b^	670.6 ± 117.1^b^	0.32	NS
Three month	573.4 ± 73.6	586.1 ± 29.0	586.7 ± 36.0	1.50	NS
Repeated measure within group ANOVA	F = 17.5 P = 0.0001	F = 11.4 P = 0.0001	F = 11.19 P = 0.0001		
Endothelial cell count.					
Pre-operative	2311.73 ± 288.0	2359.78 ± 383.2	2526.6 ± 305.4	2.36	NS
Day one	2021.20 ± 201.0	2035.05 ± 377.0	2196.4 ± 378.2	0.11	NS
Three month	1973.00 ± 537.0	1959.00 ± 503.0	2132.0 ± 482.0	0.82	NS
Repeated measure within group ANOVA	F = 4.2 P = 0.004	F = 2.20 P = NS	F = 17.5 P = 0.0001		

a: p < 0.05, b: <0.01, c: <0.001 as compared to preoperative parameter

UCVA = Uncorrected visual acuity

BCVA = Best corrected visual acuity

ANOVA = Analysis of Variance

NS = Not Significant

**Table 2:** Intra-operative oarameters

**Parameter**	**Group 1 n = 19**	**Group 2 n = 19**	**Group 3 n = 18**	**One-way ANOVA, F-Value**	**P value**	**Post hoc ANOVA, (Scheffe's test)**
Mean phaco energy (%)	0.28 ± 0.06	0.31 ± 0.06	0.38 ± 0.09	6.75	0.0024	I Vs III: p = 0.03 II Vs III: p = 0.05
Mean phaco time (min)	2.0 ± 1.0	1.8 ± 0.8	2.1 ± 0.7	0.026	--	--
Effective phaco time (sec)	33.8 ± 17.7	36.1 ± 20.4	49.5 ± 23.1	3.31	--	--
Mean total surgical time (min)	12.9 ± 3.3	15.2 ± 3.5	16.5 ± 3.6	5.06		1 Vs III: p = 0.011
OVD removal time (sec)	66.6 ± 11.2	45.1 ± 9.0	55.47 ± 6.6	26	0.0001	I Vs II: p = 0.0001 I Vs III: p = 0.003 II Vs III: p = 0.006

OVD = Ophthalmic viscosurgical device

min = minutes

sec = seconds
